# Language assessment of children with severe liver disease in a public service in Brazil

**DOI:** 10.6061/clinics/2017(06)04

**Published:** 2017-06

**Authors:** Erica Macêdo de-Paula, Gilda Porta, Ana Cristina Aoun Tannuri, Uenis Tannuri, Debora Maria Befi-Lopes

**Affiliations:** I Departamento de Fisioterapia, Fonoaudiologia e Terapia Ocupacional, Faculdade de Medicina, Universidade de Sao Paulo, Sao Paulo, SP, BR; II Unidade de Hepatologia, Instituto da Crianca, Hospital das Clinicas HCFMUSP, Faculdade de Medicina, Universidade de Sao Paulo, Sao Paulo, SP, BR; III Divisão de Cirurgia Pediatrica e Unidade de Transplante de Figado, Instituto da Crianca, Hospital das Clinicas HCFMUSP, Faculdade de Medicina, Universidade Sao Paulo, Sao Paulo, SP, BR

**Keywords:** Child Language, Language Development, Language Development Disorders, Liver Transplantation, Language Tests

## Abstract

**OBJECTIVE:**

The aim of this research was to compare language development (expressive and receptive skills) in children awaiting liver transplantation with that of children who have already undergone the surgical procedure.

**METHODS:**

An observational, descriptive, cross-sectional study was conducted with 76 children divided into groups, as follows: 31 children who were candidates for liver transplantation (Group 1; G1), 45 children who had already undergone liver transplantation (Group 2; G2), and a control group (CG) of 60 healthy, normally developing children. Health status information was gathered, and the Test of Early Language Development (TELD)-3 was used to assess language skills. Family household monthly income data were also gathered using a specific questionnaire.

**RESULTS:**

G1 had poorer language performance compared with G2 and the CG. G2 had lower language performance when compared with the CG. However, when considering the TELD-3 standard scores, G2 had scores within normal limits. The regression analysis indicated age as a risk factor for language deficits in Group 1 and family income as a risk factor for language deficits in G2.

**CONCLUSIONS:**

The results suggested that children with chronic liver disease have delays in language development. Transplanted children have linguistic performance within normal limits, but their scores tended to be lower than the CG.

## INTRODUCTION

Worldwide, the liver is the second most commonly transplanted major organ. Liver transplantation aims to restore health conditions in patients with end-stage liver diseases. It is an effective treatment with a survival rate of approximately 80% at five years after the procedure 1. Since 2008, the number of liver transplantation has increased considerably in Brazil. In 2013, Brazil performed the second highest number of liver transplants out of 30 countries, reaching a total number of 1,723 procedures. Regarding pediatric liver transplantation, in 2014, 190 liver transplantations were performed for children in Brazil. Of these, 142 were performed in the State of São Paulo 2.

Due to increasing survival rates, other aspects involved in liver transplantation are gaining importance. Researchers have focused on topics such as adherence to treatment, quality of life, the complications and consequences of long-term use of immunosuppression and possible language, cognitive and social deficits related to liver chronic diseases.

Evidence of language delay (in expressive and receptive skills) has been described previously in infants, preschoolers and children pre- and post-liver transplantation 3-8. According to the literature, the level of bilirubin and the age at the time of liver transplantation, height and weight (i.e., below average), hypoalbunemia, low levels of vitamin E, frequent hospitalizations and increased abdominal circumference can lead to pulmonary problems and feeding difficulties and are considered crucial factors for language and cognitive development in transplanted children 3,9-12.

Caudle et al. 3 observed that infants with biliary atresia who needed liver transplantation had expressive language disorders and deficits in receptive language skills. A study published in 2012 indicated that girls with biliary atresia prior to liver transplantation had greater language and cognitive deficits compared with boys 8. The level of bilirubin was the only variable that differed between the groups.

Language development has universal hallmarks that are observed in all children. However, a wide range of variability exists among individuals. Variability is present in terms of first words acquisition and production, age at which first utterances (i.e., the combination of two or more words) are produced and during the early stages of syntax development. This variability is explained by socioeconomic, family environment, emotional, psychological, cognitive and genetic aspects, and especially by the combination of these factors 13.

Although every child is unique and language development occurs at different rates in children, some milestones are expected. Specific literature on normal language development has noted that children usually begin to produce their first words near the age of one year. By their second birthday, most children have a vocabulary of approximately 50 words and start using two-word phrases. By the age of three years, speech becomes more accurate; by the age of four years, syntax stabilizes, and children are able to express their ideas and feelings with more precision rather than just talking about the world around them. At the age of five years, speech is completely understandable, and utterances can be eight or more words long. By their sixth birthday, children are able to tell and retell stories and events in a logical order and are able to express ideas using a great variety of complete sentences. Finally, when children reach seven years, they can use oral language to inform, persuade, and entertain 13,14.

There are many possible causes of language delays in children. Usually, more than one factor contributes to the delay. Some common causes are hearing impairment, neurological disorders, psychological and social factors and intellectual disabilities 15. Many children with severe liver disease experience social deprivation, cognitive deficits and, in some cases, neurological disorders 3,9-12. Most of the existing studies on liver transplantation have focused on the characterization of individuals prior to the surgical procedure. However, due to the growing survival rates of this population, there is a need to verify the effects of liver transplantation on such aspects as language development.

The present study aimed to describe the language development (expressive and receptive) of children with severe liver disease, to compare the performance of children awaiting liver transplantation with that of children who have already undergone the surgical procedure, and to identify possible risk factors for language development deficits in this population.

## METHODS

The study design was approved by the Ethics Committee for the Analysis of Research Projects (CAPPesq HCFMUSP no. 35723). Prior to their enrollment, all the participants and their respective families were informed of the purpose and procedures of the study, after which they all gave written informed consent.

One of the most important pediatric liver transplantation centers is located at Hospital das Clínicas in the city of São Paulo, Brazil. This hospital is the largest public school hospital in South America. Hospital das Clínicas performs approximately 20 pediatric liver transplants each year.

An observational, descriptive, cross-sectional study was conducted with children who had chronic liver disease and either were candidates for liver transplantation or had already been transplanted. All children under treatment at the time of the study, including those who had already undergone transplantation and those who were still waiting for the procedure, were referred by the medical team to the Division Speech and Language Pathology for language assessment. The parent of one patient did not consent; all the other patients were included in the study.

The children were divided into two groups, as follows: Group 1 (G1) comprised candidates for liver transplantation, and Group 2 (G2) comprised children who had already undergone liver transplantation. All the children were seen at the Pediatric Surgery Clinic of the Children’s Institute of Hospital das Clínicas between January 2012 and July 2014.

Patients who met the following criteria were eligible for participation: the children in G1 had be on the hospital’s waiting list for liver transplantation, and for the children in G2, the time between liver transplantation and language assessment had to be six months or more; the children’s ages were between 2 years and 7 years and 11 months; and the children could not have neurological injuries or other coexisting diseases. Three transplanted children were excluded due to fulminant hepatitis, and one child was excluded due to visual impairment that compromised the assessment. There were no exclusions after the data gathering procedures began. All children who were referred to the Division of Speech-Language Pathology were assessed.

For both groups, socioeconomic status was determined based on the family’s household monthly income, which had to be between 1,500–2,500 in the local currency. Monthly income was determined according to the average of the family’s household monthly income at the time of the language assessment. The socioeconomic data were collected by the researchers on the day of assessment.

A CG of 60 healthy children, 30 boys and 30 girls, with normal language development was recruited for comparison purposes. All the children in the CG were enrolled in a public school in São Paulo. To be included in this group, the children had to have an absence of language development deficits and/or neurologic and cognitive deficits and had to present hearing thresholds within normal limits.

For the selection of the CG, the school files of each child were consulted to verify academic performance and any record of possible deficits in development. Moreover, the teachers gave information about the children’s performance in school, and the parents answered a questionnaire about previous alterations in development and about the child’s overall health status. After this information was gathered, we excluded all children who presented any history of possible deficits and/or alterations in development. The control children were matched to the research children by age and socioeconomic status 12.

### Language Assessment

All the participants were native speakers of Brazilian Portuguese and were assessed by an experienced speech-language pathologist. The Brazilian version 14 of the Test of Early Language Development (TELD)-3 15 was used. This test assesses the language of children aged 2 years to 7 years and 11 months. The aim of the test is to provide a comprehensive measure of expressive (i.e., ability to produce language) and receptive (i.e., ability to understand language) language skills, including an evaluation of semantic and morphosyntactic aspects of language.

The TELD-3 (third version) examiner’s’ guide book gives information about the test’s reliability. According to this guide, the instrument presents high reliability for all subtests, as follows: Receptive Language (Cronbach’s alpha coefficient of 0.91), Expressive Language (Cronbach’s alpha coefficient of 0.92), and the Spoken Language ratio (Cronbach’s alpha coefficient of 0.95). The validity of TELD-3 was verified in different ways and was compared with other gold-standard tests for language assessment in children. In these comparisons, the TELD-3 presented similar results for all three subtests. When compared with tests that assess cognitive performance, the TELD-3 presented a moderate correlation (0.70) 16.

According to the test’s scoring requirements, the child received one point for each item answered correctly and received zero for each item answered incorrectly on each subtest. All the items had specific scoring criteria (how the child must perform or respond to obtain points). The points obtained were added (raw scores) and then converted into quotients using the conversion tables provided by the test.

The receptive language subtest consisted of 37 items. The instructions for this part of the test required the child to point to specific pictures showing that he/she recognized different objects and adjectives; answer questions regarding different stories; identify phrases with syntactic or semantic errors; and find words that had a semantic relationship.

The expressive language subtest consisted of 39 items. In this subtest, the child had to name pictures, tell stories, repeat increasingly complex phrases, and answer questions to demonstrate that he/she was able to produce a proper answer, formulate phrases and complete phrases with missing words.

Finally, the expressive and receptive subtest scores were combined to provide the Spoken Language ratio, which is considered an efficient indicator of oral language development.

Table 1 shows the classification of the quotation ratios. This classification was used to analyze the results of the two language sub-tests and the final measurement of Spoken Language.

The administration of the TELD-3 lasts approximately 20 minutes. All children in G1 and G2 were assessed at the Division of Pediatric Surgery and the Liver Transplantation Unit of the Children’s Institute of Hospital das Clínicas (School of Medicine, University of São Paulo, Brazil). The children were assessed individually in a quiet room on the day of their medical appointment. All the children in the CG were also assessed individually, in a room designated for this purpose at their own school.

### Clinical parameters

Clinical information (i.e., length of hospital stay, weight and height, which were then used to calculate the z-score) regarding the children’s overall health status (for G1 and G2) was obtained by analyzing medical records.

The following clinical tests were obtained on the day of the language evaluation: total bilirubin, direct bilirubin, indirect bilirubin, sodium, relationship between the activated partial thromboplastin time (APTT) of the patients and the APTT of the controls, prothrombin time, international normalized ratio (INR), gamma-glutamyl transferase (GGT), albumin, aspartate aminotransferase (AST), and alanine aminotransferase (ALTX).

### Data analysis

A descriptive analysis of quantitative data was performed using mean values and standard deviations (SDs). The assumption of normal distribution in each group was assessed with the Shapiro-Wilk test. Categorical variables are shown as frequencies and percentages.

Analysis of variance (ANOVA) was used to analyze differences in the ratio of language performance among G1, G2 and the CG. Student’s t test was used for between-group comparisons for categorical variables, considering independent samples. The Mann-Whitney test was used for between-group comparisons of quantitative data. The adopted level of significance was 5%.

Univariate logistic regression analysis was used to explore the correlation between clinical and socioeconomic variables and language performance. For this analysis, we selected 16 clinical and socioeconomic variables (gender, length of hospital stay, age, AST, ALTX, GGT, total bilirubin, direct bilirubin, indirect bilirubin, albumin, INR, APTT, sodium, z-score height, z-score weight, and family income).

Variables with *p*≤0.1 were included in the multivariate logistic regression analysis, which employed a stepwise forward likelihood ratio model.

## RESULTS

The selected sample consisted of 76 children with severe liver disease. G1 comprised 31 children, 15 boys and 16 girls, who were candidates for liver transplantation. These children had the following diagnoses: 18 had biliary atresia, 4 had cirrhosis, 4 had Alpha-1 antitrypsin deficiency, 2 had Alagille syndrome, 2 had familial intrahepatic cholestasis and 1 had glycogen storage disease Type IV.

G2 comprised 45 transplanted children (17 boys and 28 girls). The children in this group had the following pre-transplant diagnoses: 37 had biliary atresia, 2 had cirrhosis, 2 had Alpha-1 antitrypsin deficiency, 1 had Alagille syndrome, 1 had glycogen storage disease Type IV, 1 had familial intrahepatic cholestasis, and 1 had cyst sclerosing cholangitis. The average age at which the children underwent transplantation was 17 (±8.4) months, and the time between transplant and language evaluation was 43.5 (±31.1) months.

According to ANOVA, the groups were homogenous in terms of socioeconomic status (*p*=0.357), the mean household monthly income in *reais* was 1,568. 00 (±1,254) for G1, 2,063.00 (±1,318) for G2 and 1,811.00 (±869) for CG. However, the groups differed significantly in age (*p*=0.005*); the average age in years was 3.6(±1.4) for G1, 4.25(±1.4) for G2 and 4.9(±1.7) for the CG. The children in G1 were significantly younger than those in the CG: G1 *versus* G2, *p*=0.397; G1 *versus* CG, *p*=0.004*; and G2 *versus* CG, *p*=0.200.

The groups differed significantly in all the assessed language parameters. Multiple comparisons indicated that the children in G2 had expressive language performances similar to those of the children in the CG. The children in G1 had worse language performance on all the parameters compared with the children in the other two groups (Table 2).

The comparison of the clinical parameters between G1 and G2 showed that overall, G1 had a worse health status compared with G2 and when compared with the expected normal range values. However, although G2 had a better clinical health status than G1, the children were still outside the normal range values for some clinical parameters (Table 3). The normal range values presented are the standard parameters used at our hospital.

Sixteen independent variables were considered for the univariate analysis (logistic regression) for G1 and G2. Statistically significant language development indicators included the age of the liver transplantation candidates (i.e., the older the child, the higher the risk of presenting language delays) and the sodium levels and socioeconomic status of the transplanted children (i.e., the higher the sodium levels, the higher the risk of presenting language delays; Table 4).

Multivariate analysis was performed to determine whether the association of status remained as a language development indicator for the children in G2. In this analysis, only the family’s socioeconomic status remained independently associated with language development outcomes (OR=0.999; IC=0.999–1.000; *p*=0.055*); i.e., for G2, only socioeconomic status was a risk factor for poor language performance.

## DISCUSSION

Unlike the studies in the literature on the linguistic and cognitive performances of children with chronic liver disease, our study compared the language performance of children with severe liver disease to that of healthy normally developing children (i.e., the CG). Overall, our results indicated a delay in language development for children who were candidates for liver transplantation compared with a group of children who had already been transplanted and to a control group of healthy individuals. Although the children who had already been transplanted had linguistic performances within normal limits, their scores on the language test were lower than those of the control group, indicating that after transplantation, these children do not catch up with healthy children who have normal language development.

Concerning language receptive abilities, our results demonstrated that the groups differed in performance, indicating that the children in G1 and G2 had difficulties in language comprehension. According to the literature, difficulties in language comprehension have a direct impact on the ability to understand stories, instructions, long and/or complex utterances and identify different objects 17,18.

Expressive language abilities, in comparison, are necessary for children to communicate, name objects, and express needs and ideas 17,18. Our results indicated that the children in G1 had a worse performance compared with the children in G2 and the CG. Considering that language comprehension precedes language expression 18 and that the children in G2 presented worse receptive language abilities than those in the CG, we expected that the group of transplanted children would also have a worse performance on expressive language abilities compared with their healthy peers. We believe that our results may have been influenced by our sample size and by the heterogeneity of our research sample.

At this point, it is not possible to provide a solid explanation for the linguistic delay presented by G1 since the results of the regression analysis did not indicate any of the investigated clinical variables or the socioeconomic status as possible predictors of low linguistic performance. Nonetheless, we suggest that the etiology of the linguistic deficit could be associated with social deprivation, which increases with advancing age (a risk factor indicated in the results) in relation to the specific clinical factors observed in liver diseases 5.

The TELD 3 has been validated for normally developing children and for children with attention deficits and hyperactivity, learning disorders, and language and cognitive delays. The results obtained for G1 were similar to those observed in children with learning difficulties (LD) for all three parameters - expressive language (G1 - 85.68; LD - 85); receptive language (G1 - 84.48; LD - 81) and spoken language (G1 - 82.19; LD - 80) - thus confirming that this group had a language delay.

Krull et al. 5 analyzed the cognitive and linguistic development of liver transplanted children, comparing their performance with that of children with cystic fibrosis. In that case, clinical and social variables were also correlated with performance on language tests. The authors observed that the group of transplanted children had lower IQs and lower performances on language tests. The authors concluded that social deprivation and isolation did not explain the language delay observed in the transplanted group. The logistic regression of our results did not indicate a specific clinical variable or socioeconomic status that predicted the language performance of the children in G1. Studies have noted that the etiology of the linguistic deficit could be the social deprivation associated with other clinical factors in chronic liver diseases 5,18.

Until very recently, only children with liver disease of a metabolic nature who were candidates for liver transplantation were believed to have linguistic and neuropsychological deficits 19. However, the results of our study agreed with more recent work suggesting that children with biliary atresia, who are also candidates for liver transplantation, also have language delays 8,20. This result confirms the need for a language assessment follow-up for all children awaiting liver transplantation.

Stewart et al. 21 verified the cognitive development of children with chronic liver disease before one year of age and between one year and half and twelve years. The authors found that the IQ was lower for those children who had disease symptoms at an earlier age. This result suggests that metabolic alterations caused by a chronic liver disease are more harmful to the neurologic system/development during the first year of life. These data give evidence of the importance of performing liver transplantation as early as possible and confirms our finding that age is an important risk factor for language delay.

Children who are transplanted at an older age are not only exposed to the liver disease for a longer time but are also deprived of social interactions and daily routines for long periods. This fact most certainly contributes to the linguistic delay observed in these children and explains why age is a risk factor. Additionally, the fact that neural plasticity decreases with age must be taken into consideration as an explanation of why older children have lower chances of obtaining full linguistic development 22.

Although G1 was significantly younger than G2, most of the children in G2 were transplanted at an early age (before 2 years). This was not true for the children in G1 since they already had a mean age of 3.6 years and were still waiting for transplantation. Although the language test used in our study considers the age variable, the children in G1 presented a more severe health status, which may have contributed to the observed language delay.

Although the children in G2 had already been transplanted, several of their clinical parameters remained altered. This fact may have influenced the group’s performance on the TELD-3. Another fact that should be considered is that the continuous use of immunosuppressive drugs may have an impact on language development. As already known, these drugs can affect memory and logical reasoning, which are essential elements of adequate language development 23.

Our study also found that socioeconomic status had a positive correlation with lower language performance in G2. There is consistent evidence in the literature pointing to the existence of a correlation between lower socioeconomic status and linguistic deficits 16,24,25. Considering that the groups in our study had the same socioeconomic status, the results suggested the possibility that the association between the low socioeconomic status, chronic liver disease and the continuous use of immunosuppressive drugs contributed to low performance on the language tests. Posfay-Barbe et al. 20 concluded that although a significant number of children show developmental and social deficits following liver transplantation, several aspects of family functioning favor normal development, including parental functioning and parental level of education.

### Limitations and further research

It is important to highlight a few potential limitations in our study that may have undermined our conclusions. It was a single-institution study, and its findings may reflect local patient characteristics; it was not a follow-up study since the children in G1 and G2 were not the same; the patients with chronic liver disease were heterogeneous (in terms of age, diagnosis, time on the waiting list, and age at transplant); for the children in G2, the time between liver transplantation and language assessment was variable; and the sample size was small and therefore, the results cannot be generalized. Future studies should include a larger sample size, preferably with patients divided into different age groups, as there is a great difference in language development in children between the ages of 2 and 7 years.

Although most language tests can only be reliably applied to children over two years of age, future research should focus on verifying the language abilities and development of children under two years of age as they represent the majority of the population on the waiting list for liver transplantation. Patients who are transplanted after two years of age usually have metabolic disease and often present with developmental deficits, which might have influenced our results. Moreover, it would be interesting to conduct a longitudinal follow-up of transplanted patients (i.e., prior to and after liver transplantation) to effectively evaluate the development of language. Studies of this nature were not found in the current literature.

Our results demonstrated that the groups differed in performance, indicating that the children in G1 and G2 had difficulties with language comprehension and that the children in G1 had a worse performance than the children in G2 and the CG. It is not possible to provide a solid explanation for the linguistic delay presented by G1 since the results of the regression analysis did not indicate any of the investigated clinical variables or the socioeconomic status as possible predictors of low linguistic performance. Our study also found that socioeconomic status was positively correlated with lower language performance in G2.

In short, our study suggested that children with chronic liver disease present a delay in language development compared with their normally developing healthy peers. The transplanted children had linguistic performances within normal limits, but their scores on the language test tended to be lower than the scores of children in the CG, indicating that children who have undergone liver transplantation do not catch up to the healthy children with normal language development.

## AUTHOR CONTRIBUTIONS

de Paula EM collected and interpreted data and wrote the main manuscript. Porta G designed and supervised the project and edited the manuscript. Tannuri AC supervised the project and edited the manuscript. Tannuri U provided technical support and conceptual advice and approved the manuscript final version to be published. Befi-Lopes DM designed the experiment and project, supervised its analysis and edited the manuscript. All authors discussed the results and implications and commented on the manuscript at all stages.

## Figures and Tables

**Table 1:** Rating ratios provided by the TELD-3.

Quotients ratios	Classification	Category used for statistical analysis
131 – 165	Very superior	Adequate language development
121 – 130	Superior	
111 – 120	Above average	
90 – 110	Average	
80 – 89	Below average	Language development below expectations
70 – 79	Poor	
35 – 69	Very poor	

**Table 2:** Comparisons of language performance.

	Mean (±SD)			*p*-value	Multiple comparisons
	G1	G2	CG		
Receptive language	85.68 (8.84)	93.20 (14.17)	101.38 (10.87)	<0.001*	G1≠G2 *p*=0.020* G1≠CG *p*<0.001* G2≠CG *p*=0.002*
Expressive language	84.48 (11.12)	92.75 (13.37)	94.66 (8.09)	<0.001*	G1≠G2 *p*=0.004* G1≠CG *p*<0.001* G2=CG *p*=1.000
Spoken language	82.19 (9.85)	92.07 (13.71)	97.57 (8.3)	<0.001*	G1≠G2 *p*<0.001* G1≠CG *p*<0.001* G2≠CG *p*=0,031*

Legend: G1 – candidates for liver transplantation; G2 – transplanted children; CG – control group; SD – standard deviation; * - significant results; ANOVA test; post hoc analysis Bonferroni test.

**Table 3:** Comparison of clinical parameters between G1 and G2.

	Normal Range	G1 Mean (±SD)	G2 Mean (±SD)	*p*-value
AST U/L	16 – 57	320.50 (261.08)	59.22 (23.7)	<0.001*
ALT U/L	24 – 59	208.54 (147.10)	70.86 (57.94)	<0.001*
GGT U/L	8 – 59	313.04 (427.34)	113.84 (169.30)	<0.001*
Total bilirubin mg/dL	0.2 – 1	10.51 (9.23)	0.52 (0.41)	<0.001*
Direct bilirubin mg/dL	0.05 – 0.3	9.21 (8.53)	0.29 (0.49)	<0.001*
Indirect bilirubin mg/dL	0.1 – 0.6	1.29 (1.18)	0.28 (0.19)	<0.001*
Albumin g/dL	2.3 – 4.7	3.40 (0.66)	3.65 (0.75)	0.019*
INR seconds	0.9 – 1.2s	1.48 (0.82)	1.35 (0.866)	0.488
APTT	1 – 1.15	2.63 (7.17)	0.94 (0.26)	0.062
Sodium mEq/L	138 – 145	137.37 (2.24)	138.62 (2.63)	0.085
z-score weight	-	-1.28 (1.77)	-0.23 (1.55)	0.826
z-score height	-	-1.48 (1.91)	-1.49 (1.97)	0.078
Length of hospital stay (days)	-	70.8 (82.2)	97.2 (88.2)	0.105

Legend: G1 – candidates for liver transplantation; G2 – transplanted children; SD - standard deviation; AST - aspartate aminotransferase; ALT - alanine aminotransferase; GGT - gamma-glutamyl transpeptidase; INR - international normalized ratio; APTT - activated partial thromboplastin time; z-score height (observed height – mean height for age)/s.d.; z-score weight (observed weight – mean weight for age)/s.d.; U/L - units per liter; mg/dL - milligrams per deciliter; g/dL - grams per deciliter; s – seconds; mEq/L – milliequivalents per liter; * - significant result; Mann-Whitney test.

**Table 4:** Logistic regression (univariate analysis) of independent variables for the spoken language ratio.

Variables	G1		G2	
	OR (CI 95%)	*p*-value	OR (CI 95%)	*p*-value
Gender (F/M)	0.455 (0.089-2.318)	0.343	0.629 (0.182-2.178)	0.465
Length of hospital stay	1.066 (0.992-1.020)	0.418	1.001 (0.995-1.008)	0.672
Age	1.075 (1.00-1.156)	0.050*	1.002 (0.978-1.028)	0.848
AST	1.001 (0.009-1.005)	0.532	0.997 (0.983-1.012)	0.691
ALTX	1.002 (0.995-1.009)	0.603	0.999 (0.988-1.009)	0.812
GGT	1.000 (0.998-1.002)	0.875	0.999 (0.995-1.003)	0.625
Total bilirubin	1.007 (0.916-1.108)	0.879	0.898 (0.212-3.801)	0.884
Direct bilirubin	1.008 (0.909-1.117)	0.833	0.763 (0.206-2.822)	0.685
Indirect bilirubin	1.048 (0.502-2.190)	0.900	0.285 (0.010-8.024)	0.461
Albumin	0.477 (0.113-2.022)	0.315	1.577 (0.535-4.645)	0.408
INR	1.955 (0.392-9.756)	1.955	0.323 (0.012-8.652)	0.500
APTT	0.890 (0.661-1.199)	0.444	4.331 (0.094-199.612)	0.453
Sodium	1.379 (0.899-2.116)	0.141	0.731 (0.556-0.962)	0.025*
z-score height	0.938 (0.590-1.491)	0.786	0.964 (0.678-1.371)	0.837
z-score weight	0.910 (0.549-1.507)	0.713	1.151 (0.741-1.787)	0.532
Family’s income	1.001 (0.999-1.002)	0.330	0.999 (0.999-1.000)	0.089*

Legend: G1 – candidates for liver transplantation; G2 – transplanted children; SD - standard deviation; AST - aspartate aminotransferase; ALT - alanine Aminotransferase; GGT - gamma-glutamyl transpeptidase; INR - international normalized ratio; APTT - activated partial thromboplastin time; z-score height (observed height – mean height for age)/s.d.; z-score weight (observed weight – mean weight for age)/s.d.; OR – odds ratio; CI – confidence interval; * - significant result.
